# Engineered prebiotic microcapsules Co-Encapsulating berberine and curcumin Elicit multi-synergistic therapy for ulcerative colitis

**DOI:** 10.1016/j.mtbio.2026.102778

**Published:** 2026-01-07

**Authors:** Huanyu Li, Chuanyu Zhang, Ziwei Yang, Yifan Li, Dan Liu, Yanan Zhang, Lingmin Zhang, Ning Wang, Mingxin Zhang, Mingzhen Zhang, Zhaoxiang Yu, Xueyong Wei, Yujie Zhang

**Affiliations:** a Institute of Translational Medicine, School of Basic Medical Sciences, Xi'an Jiaotong University, Xi'an, Shaanxi, 710061, China; b Department of Laboratory Medicine, Chongqing Center for Clinical Laboratory, Chongqing Academy of Medical Sciences, Chongqing General Hospital, School of Medicine, Chongqing University, Chongqing, 401147, China; c School of Instrument Science and Technology, Xi'an Jiaotong University, Xi'an, Shaanxi, 710049, China; d State Key Laboratory for Manufacturing Systems Engineering, Xi'an Jiaotong University, Xi'an, Shaanxi, 710049, China; e Second Clinical Medical College, Shaanxi University of Chinese Medicine, Xianyang, Shaanxi, 712046, China; f State Key Laboratory of Holistic Integrative Management of Gastrointestinal Cancers and National Clinical Research Center for Digestive Diseases, Xijing Hospital of Digestive Diseases, Fourth Military Medical University, Xi'an, Shaanxi, 710032, China; g The First Affiliated Hospital of Xi'an Medical University, Xi'an, Shaanxi, 710077, China; h Department of Anesthesiology, The First Affiliated Hospital of Xi'an Jiaotong University, Xi'an, Shaanxi, 710061, China; i Department of Anaesthesiology, The Second Affiliated Hospital, Xi'an Jiaotong University, Xi'an, Shaanxi, 710004, China

**Keywords:** Berberine, Curcumin, Prebiotic microcapsules, Ulcerative colitis, TNF signaling pathway, AGE-RAGE signaling pathway

## Abstract

**Background:**

Ulcerative colitis (UC) is a common type of inflammatory bowel disease, where the vicious cycle of inflammation and oxidative stress poses a major challenge in its treatment, and existing therapies have limitations. Berberine (BBR) and curcumin (CUR) have the potential for synergistic treatment of UC, but this potential has not been verified in UC. Additionally, both BBR and CUR suffer from poor water solubility and low bioavailability. This study aims to construct prebiotic microcapsules (BBR/CUR@MC) for the co-delivery of BBR and CUR and explore their therapeutic mechanism in UC.

**Methods:**

Network pharmacology was used to predict the targets and pathways of BBR and CUR in UC. BBR/CUR@MC was prepared using microfluidic electrospray technology, and its colon targeting and biocompatibility were evaluated through *in vivo* experiments. In a Dextran sulfate sodium (DSS)-induced UC mouse model, the therapeutic effect was assessed using multiple indicators, and the mechanism of action was explored by transcriptome analysis.

**Results:**

Network pharmacology showed that BBR and CUR can exert therapeutic effects on UC through synergistic regulation of TNF and AGE-RAGE signaling pathways. The successfully constructed BBR/CUR@MC had good colon targeting and biocompatibility. In the mouse colitis model, oral administration of BBR/CUR@MC inhibited ADAM17 in the TNF signaling pathway and MAPK11/13, COL4A1, and COL1A1 in the AGE-RAGE signaling pathway, thereby downregulating pro-inflammatory cytokines such as TNF-α and IL-1α, upregulating IL-10, scavenging ROS, significantly alleviating colonic inflammation and repairing the intestinal barrier in mice, with a therapeutic effect superior to that of single-drug microcapsules and 5-ASA.

**Conclusion:**

BBR/CUR@MC can exert synergistic anti-inflammatory and antioxidant effects in UC treatment by regulating TNF and AGE-RAGE signaling pathways, providing a new multi-mechanism therapeutic strategy for UC.

## Graphical abstract

Current UC therapies struggle with the inflammation-oxidative stress vicious cycle and low bioavailability of natural drugs. This study develops an innovative colon-targeted co-delivery system, BBR/CUR@MC, fabricated via microfluidic electrospray, synergistically regulating the TNF signaling pathway (anti-inflammatory), scavenging ROS while inhibiting AGE-RAGE signaling pathway targets (antioxidant), and repairing the intestinal mechanical barrier.

**Figure:**
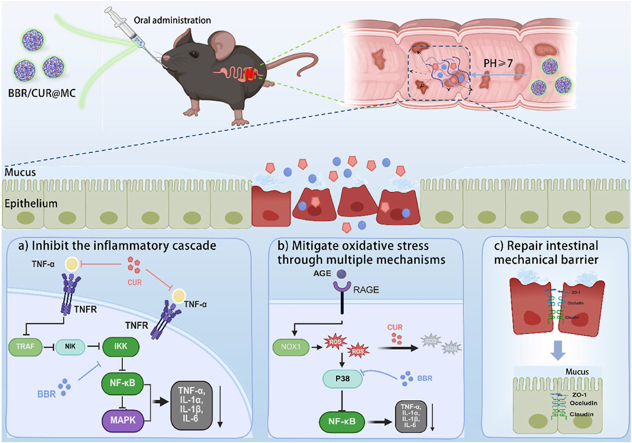

## 1 Introduction

Ulcerative colitis (UC), a prevalent form of inflammatory bowel disease (IBD), is clinically hallmarked by recurrent abdominal pain, diarrhea, and mucopurulent bloody stools, profoundly impairing patient quality of life [1]. Its incidence has risen steadily over the past two decades, imposing a substantial burden on global healthcare systems [2,3]. The pathogenesis of UC initiates with intestinal barrier disruption, enabling microbial antigens to infiltrate and activate immune cells such as macrophages. These cells subsequently release proinflammatory cytokines and recruit neutrophils, which generate excessive reactive oxygen species (ROS) that exacerbate epithelial damage while activating pathways like NF-κB and MAPK—creating a vicious cycle of inflammation and oxidative stress that ultimately leads to persistent intestinal mucosal injury [[4], [5], [6]]. Current UC therapies largely rely on aminosalicylates, glucocorticoids, immunomodulators, and biologics, which alleviate symptoms by suppressing immune responses and inflammation [7]. However, their long-term use is plagued by adverse effects and high recurrence rates, limiting their clinical utility [8,9]. Notably, within the intricate signaling network involving excessive ROS and immune-mediated inflammation, targeting a single component yields limited therapeutic benefit [10]. Thus, multimodal interventions to restore intestinal homeostasis have emerged as a promising paradigm in IBD management [11].

Berberine (BBR), an active isoquinoline alkaloid derivative isolated from the rhizome of *Coptis chinensis*, exhibits diverse pharmacological properties including antibacterial, anti-inflammatory, and antioxidant activities [12,13]. In traditional Chinese medicine, it has been employed for centuries to treat gastrointestinal infections, diarrhea, and inflammatory conditions. Modern research further indicates that BBR modulates gut microbiota composition, underscoring its potential in UC therapy [14,15]. Curcumin (CUR), the principal bioactive polyphenol in *Curcuma longa*, was historically used as a culinary spice. Contemporary pharmacological studies reveal its extensive regulatory effects on the gastrointestinal system, encompassing gut microbiota modulation, intestinal barrier enhancement, and attenuation of intestinal inflammation and oxidative stress—supporting its promise as an adjuvant therapeutic agent for gastrointestinal disorders [[16], [17], [18]]. Preclinical evidence demonstrates that BBR and CUR act synergistically, exerting therapeutic value in bacterial infections, inflammatory diseases, and beyond [19,20]. However, their combined mechanism of action in UC remains elusive [21]. Notably, both BBR and CUR suffer from poor aqueous solubility and low bioavailability, hindering their clinical translation. Thus, developing efficient, controllable targeted delivery systems for co-administering BBR and CUR holds significant implications for UC treatment.

An optimal UC drug delivery system should precisely deliver therapeutic doses to inflamed colonic sites, minimizing non-specific exposure to the broader intestine and systemic circulation. In prior work, our group utilized microfluidic and electrospray technologies to encapsulate the small molecule pterostilbene (PSB) within prebiotic microcapsules (MCs). This carrier enabled colon-specific PSB delivery, reducing gastric degradation, while the microcapsules’ wrinkled surface prolonged colonic retention—markedly enhancing therapeutic efficacy [22]. These findings provide a rationale for using MCs as a colon-targeted delivery vehicle for BBR and CUR.

**Fig. 1:**
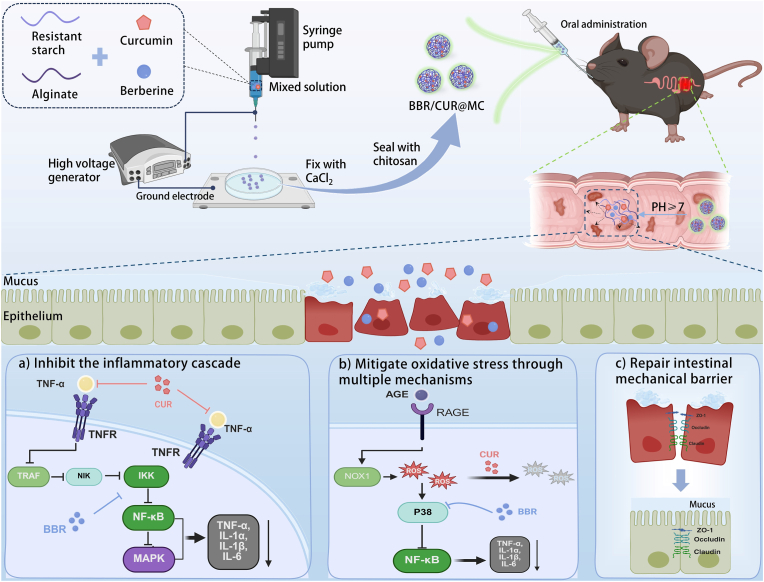
Schematic diagram of the preparation and potential therapeutic mechanism of BBR/CUR@MC. The preparation process is as follows: resistant starch (RS), alginate (Alg), berberine (BBR), and curcumin (CUR) were mixed to form a solution, which was then transferred to a syringe. After being formed into droplets via microfluidic electrospray, the droplets underwent sequential steps of CaCl_2_ solidification and chitosan encapsulation, finally obtaining BBR/CUR@MC. In the UC mouse model, after oral administration of BBR/CUR@MC, BBR and CUR were released in the alkaline intestinal environment, and colonic inflammation was alleviated through the following mechanisms: a) multi-target regulation of the TNF signaling pathway to "top-down" inhibit the cascade inflammatory response of UC. b) direct scavenging of ROS and inhibition of targets such as MAPK11/13 in the AGE-RAGE signaling pathway to achieve "full-cycle" antioxidant effect. c) restoration of intestinal tight junction proteins and mucus secretion, thereby effectively repairing the integrity of the intestinal mechanical barrier.

Herein, we employed microfluidic electrospray technology to co-encapsulate BBR and CUR into MCs, successfully constructing the BBR/CUR@MC delivery system (Fig. 1). This system enables co-targeted delivery of BBR and CUR to the colon, protecting them from gastric degradation, with demonstrated biocompatibility. Network pharmacology predictions suggested that BBR and CUR may synergistically regulate TNF and AGE-RAGE signaling pathways to exert therapeutic effects. Validation in a Dextran sulfate sodium (DSS)-induced murine colitis model confirmed that oral BBR/CUR@MC acts through dual mechanisms: first, by targeting the TNF signaling pathway to suppress inflammatory cascades. Second, by scavenging ROS and regulating the AGE-RAGE signaling pathway targets to achieve "full-cycle" antioxidant activity. Together, these mechanisms disrupt the inflammation-oxidative stress vicious cycle, repairing the intestinal barrier and alleviating colitis symptoms. In summary, this study establishes a novel paradigm for the combined application and targeted delivery of natural products, offering a potential multimodal therapeutic strategy for UC that may inform future clinical interventions.

## 2 Materials and methods

### 2.1 Materials

BBR and CUR were purchased from Shanghai Macklin Biochemical Technology Co., Ltd. Sodium alginate (Alg) was obtained from Alta Aesar. Resistant starch (RS) was synthesized by Yuanyebio Co., Ltd. CaCl_2_ was purchased from Sigma-Aldrich. Chitosan, acetic acid, and hydrochloric acid were obtained from Aladdin. Lumisphere monodisperse fluorescent microspheres were purchased from Tianjin Baseline Chromatography Technology Research Center. Near-infrared fluorescently labeled polystyrene microspheres were obtained from Suzhou Nano-Micro Technology Co., Ltd. DCFH-DA and DHE fluorescent dyes were purchased from Beyotime Biotechnology Co., Ltd. DSS was obtained from MP Biomedicals (Santa Ana, California, USA). The remaining detection kits were purchased from Nanjing Jiancheng Bioengineering Institute.

### 2.2 Prediction of potential synergistic therapeutic pathways of BBR and CUR in UC

Firstly, the targets of BBR and CUR were predicted through the SwissTargetPrediction database (http://www.swisstargetprediction.ch/). Using "ulcerative colitis" as the search term, disease-related targets were obtained from the GeneCards database (https://www.genecards.org/) and DisGeNET database (https://www.disgenet.org/), and target genes with a relevance score ≥2 were selected for subsequent studies. Then, the Omicstudio tool (https://www.omicstudio.cn/tool) was used to obtain the intersection targets of BBR, CUR and UC, as well as the exclusive targets of the two in UC treatment (where the exclusive targets of BBR refer to the part of the common targets of BBR and UC that do not overlap with CUR, and the exclusive targets of CUR refer to the part of the common targets of CUR and UC that do not overlap with BBR). Subsequently, the above two types of exclusive targets were submitted to the STRING database (https://www.string-db.org/), respectively, to construct a protein-protein interaction (PPI) network. Finally, the DAVID bioinformatics resource (https://david.ncifcrf.gov/) was used for systematic exploration of the biological significance of the previously predicted potential targets, and the OmicShare tool (https://www.omicshare.com/) was used to visually display the main signaling pathways of BBR and CUR in the treatment of UC.

### 2.3 Preparation of BBR/CUR@MC

Using a modified method based on previous work [22]^,^ BBR and CUR (1:1 ratio) were homogenously dispersed in a solution of 2.0 % (w/v) alginate and 2.0 % (w/v) RS. The dispersion was loaded into a syringe, and microcapsules were fabricated via electrohydrodynamic jet printing. A capillary (150–270 μm inner diameter) was positioned 10 cm above the collector, with an 8–12 kV electrostatic potential applied. Droplets were collected in 3.0 % (w/v) CaCl_2_ for 30-min cross-linking with Ca^2+^. Subsequently, microcapsules were incubated in 1.0 % (w/v) chitosan (dissolved in 1.0 % (w/v) acetic acid) for 30 min to form an outer chitosan layer. After three washes with deionized water, they were freeze-dried for 24 h to obtain the final product.

### 2.4 Characterization of BBR/CUR@MC

The morphologies of BBR@MC, CUR@MC, and BBR/CUR@MC were observed using an optical microscope (Olympus) and a scanning electron microscope (Hitachi). Green and red fluorescently labeled polystyrene microspheres (2.0 % v/v) were added during the preparation of BBR@MC, CUR@MC, and BBR/CUR@MC. Fluorescent images were captured using a confocal microscope (Nikon) to analyze the core-shell structures of the microcapsules.

### 2.5 Encapsulation efficiency and drug loading rate of BBR/CUR@MC

5 mg of freeze-dried BBR/CUR@MC was immersed in 20 mL of PBS (pH = 7.4) with oscillation (200 rpm, 37 °C, 24 h). Then, 700 μL of supernatant was retrieved to measure the drug concentration. The encapsulation efficiency (EE) and drug loading rate (DLR) were calculated using the following formulas.

(1) $$\text{EE}\%=\frac{a\text{mount}\;\text{of}\;\text{drug}\;\text{free}}{a\text{mount}\;\text{of}\;\text{drug}\;\text{total}}\times 100\%$$

(2) $$D\text{LR}\%=\frac{a\text{mount}\;\text{of}\;\text{drug}\;\text{loaded}}{a\text{mount}\;\text{of}\;\text{BBR}/\text{CUR}@\text{MC}}\times 100\%$$

### 2.6 In vitro drug release of BBR/CUR@MC

To simulate the characteristics of the drug release under different pH conditions, 5 mg of freeze-dried BBR/CUR@MC was immersed in 20 mL of SCF, SIF, and SGF with oscillation (200 rpm, 37 °C, 24 h), respectively. Then, 700 μL of supernatant was retrieved to measure the drug concentration. The drug release rate was calculated by the following formula.

(3) $$\text{Drug}\;\text{release}\;\text{rate}=\frac{\text{drug}\;\text{concentration}\times \text{volume}}{\text{total}\;\text{amou}\text{nt}\;\text{of}\;\text{the}\;\text{drug}}\times 100\%$$

### 2.7 Animals

Eight-week-old male C57BL/6 mice were obtained from the Laboratory Animal Center of Xi'an Jiaotong University (Shaanxi, China). All animal experiments complied with the university's laboratory animal care guidelines and were approved by its Institutional Animal Care and Use Committee (IACUC).

### 2.8 Biodistribution of BBR/CUR@MC

MC and BBR/CUR@MC were fluorescently labeled with Nile blue chloride-tagged polystyrene microspheres (5.0 % v/v). Healthy mice were randomly divided into 10 independent groups (3 mice per group), corresponding to two formulations (MC and BBR/CUR@MC) and five post-gavage time points (1 h, 3 h, 6 h, 12 h, 24 h). Mice in each group received 100 mg/kg of the corresponding fluorescently labeled formulation via gavage. *In vivo* imaging was performed at different time points using the VISQUE® InVivo Smart-LF system, and the average fluorescence intensity of each group was recorded. At each time point, 3 mice were euthanized after *in vivo* imaging, and their heart, livers, spleens, lungs, kidneys, and entire digestive tracts were collected for *ex vivo* fluorescent imaging. The average fluorescence intensity of each tissue was measured to evaluate the *in vivo* distribution of BBR/CUR@MC.

### 2.9 Biocompatibility of BBR/CUR@MC

Mice were randomly divided into two groups: the control group and the BBR/CUR@MC group. The control group received normal saline by gavage, and the BBR/CUR@MC group was gavaged with 100 mg/kg/day of BBR/CUR@MC. All mice were treated daily for 5 consecutive days and euthanized 7 days after the last dose. Daily body weight was recorded. Post-euthanasia, major organs (heart, liver, spleen, lung, kidney, colon) and blood samples were collected. H&E was used to evaluate the tissue morphology and structure of the organs, and the blood was used for routine tests and biochemical analysis.

### 2.10 Therapeutic efficacy of BBR/CUR@MC in UC

Thirty male C57BL/6 mice were randomly divided into 6 groups (5 mice per group) and acclimatized for 7 days before model induction. All groups received baseline treatment for the first 7 days, followed by daily gavage interventions starting from day 8: Control group: Normal diet and water throughout the experiment, with normal saline gavage from day 8. The other five groups: DSS group, 5-ASA group, BBR@MC group, CUR@MC group, BBR/CUR@MC group): Administered 2.5 % DSS in drinking water for the first 7 days, then gavaged with normal saline, 5-ASA (100 mg/kg), BBR@MC (100 mg/kg), CUR@MC (100 mg/kg), or BBR/CUR@MC (100 mg/kg) from day 8, respectively.

During the experiment, fecal characteristics, body weight, fecal occult blood, and disease activity index (DAI) were recorded daily (DAI scoring criteria refer to Supplementary Table 1). Before euthanasia, colonic lesions were observed via endoscopy and scored using the Mouse Endoscopic Index of Colitis Severity (MEICS, Supplementary Table 2). On day 13, all mice were euthanized. Distal colon and spleen tissues were collected, colon length was measured and photographed, and colon tissue samples were segmented, fixed in tissue fixative, or stored at −80 °C. H&E staining was performed on colon tissues, and histological scoring was conducted according to Supplementary Table 3.

### 2.11 RT-qPCR of Colon tissues

15–20 mg tissue was weighed into 1.5 mL tubes, and immediately added 600 μL ice-cold lysis buffer. Homogenization was done using a microelectric homogenizer, followed by pipetting 8–10 times. After 3–5 min at ambient temperature, samples were centrifuged at 14,000 × *g* for 2 min, and the supernatant was transferred. RNA yield and quality were checked via Nanodrop (DS-11FX+, DeNovix). Total RNA was reverse-transcribed to cDNA using BeyoRTⅢ Premix and GDNA EZeraser (Beyotime). RT-qPCR with Roche SYBR Green Master Mix quantified target mRNA, and the relative transcription level was calculated via 2^−ΔΔCt^. The primary information was presented in Supplementary Table 4.

### 2.12 Immunofluorescence

Colonic tissues were paraffin-embedded, sectioned, dewaxed, and dehydrated with gradient ethanol. The samples were blocked with 2 % BSA at 37 °C for 30 min, incubated with diluted primary antibodies at 4 °C overnight, washed thrice with PBS, and then mounted and imaged.

### 2.13 Transcriptomics

Total RNA was isolated with TRIzol (Thermo Fisher, 15596018) and validated by NanoDrop ND-1000 and Bioanalyzer 2100 (concentration > 50 ng/μL, RIN > 7.0, total > 1 μg). PolyA mRNA was enriched using oligo(dT) beads (cat. 25–61005), fragmented at 94 °C for 5–7 min with a magnesium kit (cat.E6150S), reverse-transcribed to cDNA, and second-strand cDNA synthesized with dUTP. After the end repair and adapter ligation, the products were purified with beads. UDG digestion and PCR (95 °C 3 min; 8 cycles: 98 °C 15 s, 60 °C 15 s, 72 °C 30 s; 72 °C 5 min) generated strand-specific libraries (insert 300 ± 50 bp). Sequencing was done on Illumina Novaseq™ 6000 (PE150).

### 2.14 Determination of ADAM17, TNFAIP3, MAPK11, and COL1A1

The kit was equilibrated at room temperature for 30 min before the ELISA experiment. To reconstitute the lyophilized standard, 150 μL of standard diluent was added and vortexed. Gradient dilution was then performed using five 1.5 mL centrifuge tubes, each holding 150 Plate preparation involved loading blank wells with chromogenic agents A and B and stop solution, mixing 50 μL of diluted standard and biotin-antigen working solution, adding 50 μL of diluent and working solution to zero-adjustment wells, and mixing 50 μL of test sample with the working solution. Seal the plate, incubate at 37 °C for 30 min, then wash five times with 25-fold diluted wash buffer (aspirate, fill, 30-s incubation, shake dry). Add 50 μL avidin-HRP to each well, incubate at 37 °C for 30 min, and wash. Add 50 μL each of chromogenic reagents A and B, react at 37 °C in the dark for 10 min (adjustable), and terminate with 50 μL stop solution. Measure absorbance at 510 nm against the blank well; calculate sample concentrations via standard curve using the standards' OD values.

### 2.15 Statistical methods

All results were analyzed with GraphPad Prism 8.3. Intergroup comparisons used Student's t-test, data expressed as mean ± SD (Standard deviation). Significance: *∗P* < 0.05, *∗∗P* < 0.01, *∗∗∗P* < 0.001; "ns" for non-significance.

## 3 Results

### 3.1 Prediction of potential synergistic effects of BBR and CUR in the treatment of UC

**Fig. 2:**
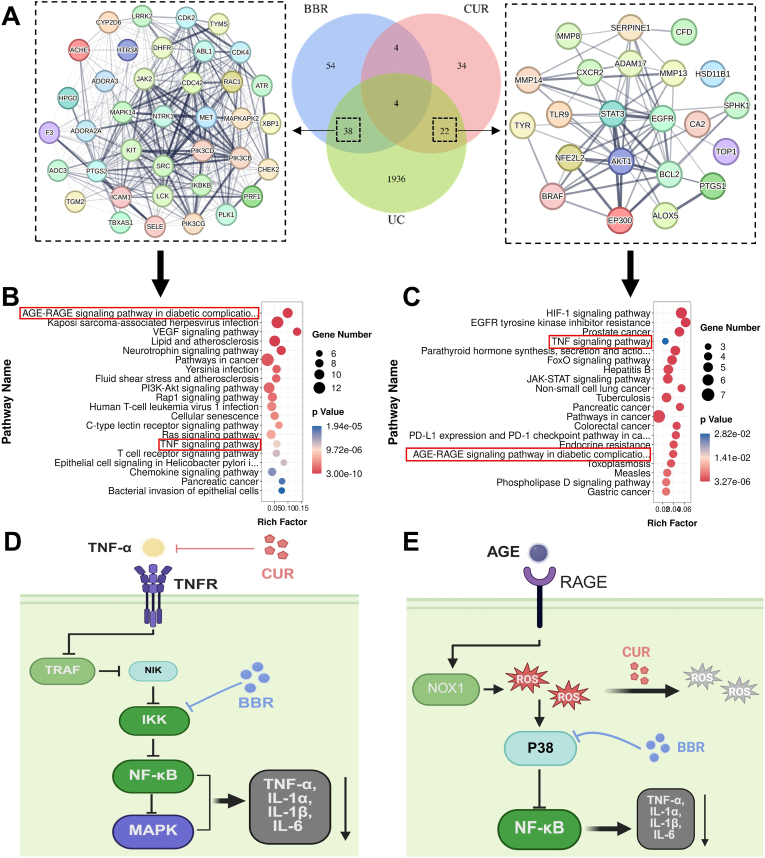
Prediction and schematic diagram of potential synergistic effects of BBR and CUR in UC treatment. (A) Screening of exclusive targets of BBR and CUR in UC and construction of PPI networks for these exclusive targets. (B) Bubble plot of KEGG enrichment analysis for BBR's exclusive targets. (C) Bubble plot of KEGG enrichment analysis for CUR's exclusive targets. (D) Schematic diagram showing that BBR and CUR inhibit inflammatory responses through synergistic regulation of the TNF signaling pathway. (E) Schematic diagram showing that BBR and CUR inhibit oxidative stress through synergistic regulation of the AGE-RAGE signaling pathway.

To investigate whether BBR and CUR exert synergistic effects in UC treatment and the underlying mechanisms, we conducted preliminary predictions and analyses via network pharmacology. First, potential targets of BBR and CUR were retrieved from the SwissTargetPrediction database. Using "ulcerative colitis" as the keyword, 2000 disease-related targets with a relevance score ≥2 were screened from the GeneCards and DisGeNET databases, and the intersection of drug targets and disease targets was identified. Analysis (Fig. 2A) showed that BBR acted on UC through 42 targets, while CUR had 26 potential therapeutic targets for UC, with only 4 common targets. Since the combined effect of the same dose on these common targets is at most equivalent to or weaker than that of double the dose of a single drug, it suggests that common targets are not the main contributors to synergistic effects. Therefore, we further explored the synergistic mechanism through the exclusive targets of BBR and CUR: BBR had 38 exclusive therapeutic targets for UC, and CUR had 24 exclusive targets. Protein-protein interaction (PPI) networks of these exclusive targets were constructed using the STRING database (Fig. 2A), and KEGG enrichment analysis was performed (Fig. 2B and C). The key finding is that both can exert therapeutic effects in UC by regulating the TNF signaling pathway and the AGE-RAGE signaling pathway in diabetic complications.

Based on network pharmacological analysis, in the TNF signaling pathway, CUR acts as an upstream blocker, targeting ADAM17 to inhibit the synthesis of TNF-α and its binding to receptors, thereby reducing the initiation of inflammation at the source. BBR targets IKBKB and MAPK14 to inhibit the cascade activation of NF-κB and MAPK pathways, blocking the amplification of downstream inflammatory responses. The combination of the two forms a "top-down" multi-target synergy through the TNF signaling pathway, collectively reducing the production of pro-inflammatory factors such as TNF-α, IL-1α, and IL-6, demonstrating the potential of synergistic anti-inflammatory therapy for UC (Fig. 2D). In the AGE-RAGE signaling pathway, CUR can directly neutralize ROS produced by this pathway through phenolic hydroxyl groups, alleviating oxidative damage. Although BBR has no direct ROS-scavenging effect, it can target and inhibit MAPK14, indirectly exerting antioxidant effects by negatively feedback-blocking upstream pro-oxidative signals (Fig. 2E). The combination of BBR and CUR achieves full-cycle reduction of oxidative damage through the dual mechanism of "scavenging existing ROS and inhibiting new ROS production," more thoroughly breaking the "oxidative stress-inflammation" vicious cycle (Fig. 2E).

### 3.2 Preparation and characterization of BBR/CUR@MC

**Fig. 3:**
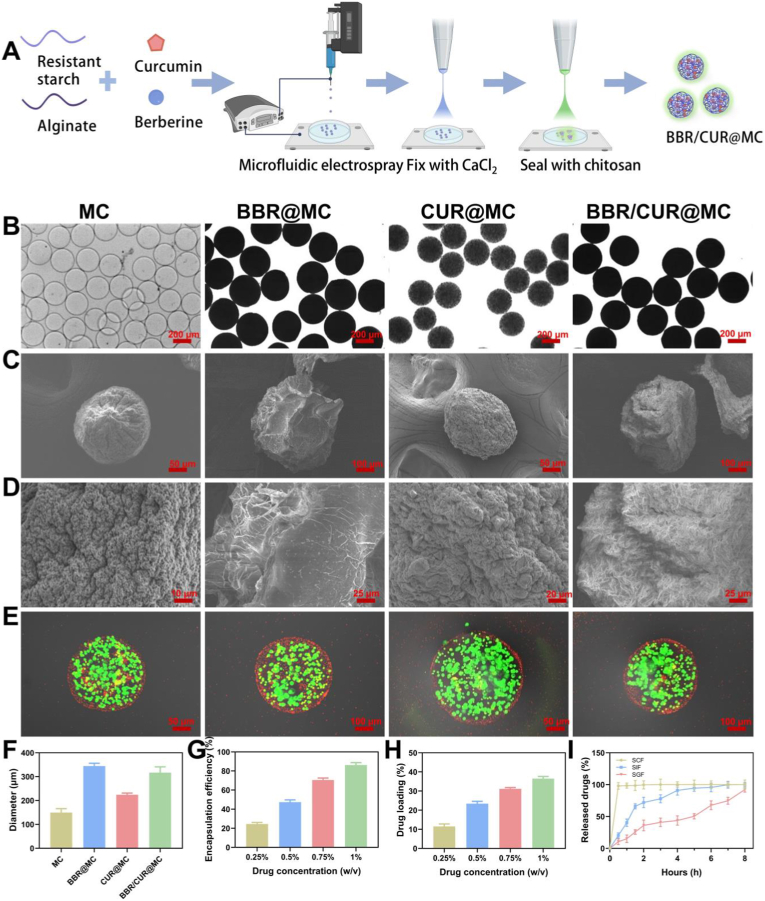
Preparation and characterization of BBR/CUR@MC. (A) Schematic of the synthesis of BBR/CUR@MC. (B–E) Morphological images of MC, BBR@MC, CUR@MC, and BBR/CUR@MC under an optical microscope (B), scanning electron microscope (C, D), and confocal microscope (E). (F) The average particle size of MC, BBR@MC, CUR@MC, and BBR/CUR@MC. (G) Encapsulation efficiency of BBR/CUR@MC at different initial drug concentrations. (H) Drug loading efficiency of BBR/CUR@MC at different initial drug concentrations. (I) Cumulative PSB release from PSB@MC at different pH conditions. (SGF: simulated gastric fluid pH = 1.2, SIF: simulated intestinal fluid pH = 6.8, and SCF: simulated colonic fluid pH = 7.4). (n = 3).

An ideal drug delivery system for UC treatment should target and deliver drugs to the colonic site at effective doses to minimize non-specific exposure to the entire intestine and the whole body. Therefore, we constructed prebiotic microcapsules (BBR/CUR@MC) for co-delivery of BBR and CUR to reduce their degradation in the stomach and achieve intestinal targeted delivery. Referring to previous methods (Fig. 3A), the mixed solution of BBR and CUR was dispersed in a mixture of 2.0 % (w/v) alginate (Alg) and 2.0 % (w/v) RS, and then the above mixture was injected into a capillary and formed into droplets via microfluidic electrospray. Since the initial loose droplet structure easily caused drug leakage, the droplets were collected in 3.0 % (w/v) calcium chloride solution, and the microcapsules were solidified through rapid cross-linking of Ca^2+^ with Alg to achieve tight encapsulation of BBR and CUR (Fig. 3A). Finally, chitosan (CS) and Alg formed an outer coating on the microcapsule surface through electrostatic interaction, resulting in BBR/CUR@MC, which can further reduce drug degradation in acidic environments (Fig. 3A). BBR@MC and CUR@MC were prepared using the same method, except that a single BBR solution or a single CUR solution was dispersed in the Alg/RS mixture in the initial step.

Subsequent characterization of the microstructure of the prepared microcapsules showed that under an optical microscope, MC, BBR@MC, CUR@MC, and BBR/CUR@MC all appeared as uniformly sized, highly dispersed spheres (Fig. 3B). The results of the scanning electron microscopy (SEM) revealed that the surface of MC was relatively smooth, while the surfaces of drug-loaded BBR@MC, CUR@MC, and BBR/CUR@MC had obvious wrinkles, and the wrinkles on the BBR/CUR@MC surface were the most obvious (Fig. 3C and D). Confocal microscopy images confirmed that all prebiotic microcapsules formed a typical core-shell structure, with red fluorescently labeled CS as the shell and green fluorescently labeled Alg/RS as the core (Fig. 3E). The particle sizes of MC and various drug-loaded microcapsules were determined and statistically analyzed via SEM. Results showed that the average particle size of unloaded MC was approximately 150 ± 16 μm, whereas after loading drugs individually or jointly, the particle sizes of the microcapsules all exhibited varying degrees of increase. Among them, the average particle size of BBR@MC was about 340 ± 12 μm, that of CUR@MC was roughly 220 ± 7 μm, and the average particle size of BBR/CUR@MC was around 310 ± 24 μm (Fig. 3F). Subsequently, we investigated the relationship between the encapsulation efficiency (EE%) and drug loading rate (DLR) of BBR/CUR@MC and the initial concentration of BBR/CUR. As depicted in Fig. 3G and H, when the initial concentration of BBR/CUR was increased from 0.25 % to 1 %, the mean EE of BBR/CUR@MC rose from 24.5 ± 1.6 % to 86.2 ± 2.5 %, while the mean DLR increased from 11.5 ± 1.3 % to 36.5 ± 1.1 %. These results demonstrate that the EE and DLR of BBR/CUR@MC exhibit a positive correlation with the initial concentration of BBR/CUR. These results indicated that BBR/CUR@MC, a prebiotic microcapsule capable of co-delivering BBR and CUR, was successfully prepared.

### 3.3 Verification of colon targeting and retention capacity of BBR/CUR@MC

The pH sensitivity of BBR/CUR@MC enables its targeted release in the intestine after oral administration. Therefore, this property was tested. In simulated gastric fluid (SGF), the release was slow, with less than 50 % of the cumulative release amount after 5 h; while in simulated colonic fluid (SCF), the release was rapid, with the cumulative release amount exceeding 95 % within half an hour (Fig. 3I). The BBR/CUR@MC attain colon targeting and prolonged retention via their core materials [22]. Alg forms a stable Ca^2+^-crosslinked hydrogel matrix to encapsulate bioactives. RS resists upper gastrointestinal degradation and confers sustained-release properties. CS forms a pH-responsive core-shell structure with Alg via electrostatic interactions—resisting gastric degradation in acidic conditions, enhancing intestinal mucosal adhesion through its antibacterial activity, and triggering microcapsule swelling for site-specific release in the alkaline colon as Alg-Ca^2+^ crosslinks dissociate and CS protonation diminishes.

**Fig. 4:**
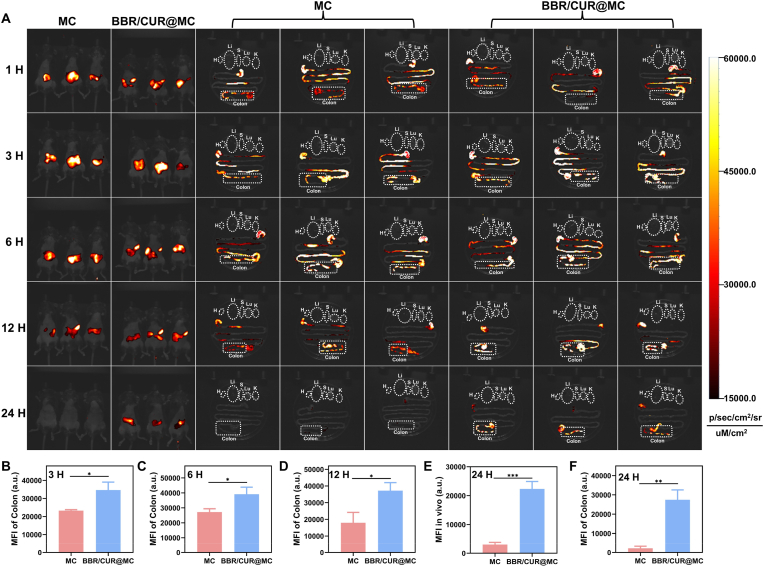
Colon targeting and retention efficacy of BBR/CUR@MC. (A) Fluorescence imaging of *in vivo* and *ex vivo* organs (liver: Li, lung: Lu, kidney: K, heart: H, spleen: S, and colon) at different time points after oral administration of fluorescently labeled MC and BBR/CUR@MC. (B–D) Statistical analysis of the average fluorescence intensity of *ex vivo* colon tissues at 3 h (B), 6 h (C), and 12 h (D). (E–F) Statistical analysis of the average fluorescence intensity *in vivo* (E) and in isolated colon tissues (F) at 24 h (n = 3; *∗P* < 0.05, *∗∗P* < 0.01, *∗∗∗P* < 0.001).

To verify the colon targeting and retention capacity of BBR/CUR@MC, healthy mice were gavaged with 100 mg/kg fluorescently labeled MC or BBR/CUR@MC, and the *in vivo* distribution of the label and targeting effect on major organs were dynamically monitored by a near-infrared small animal imager at 1 h, 3 h, 6 h, 12 h, and 24 h post-gavage. As shown in Fig. 4A, both MC and BBR/CUR@MC were significantly enriched in the colon, with no obvious accumulation in other detected organs (heart, liver, spleen, lung, kidney, and so on), confirming that MC can effectively protect drugs from gastric degradation and had highly specific colon targeting. Further *in vivo* and *ex vivo* fluorescence quantitative analysis showed that the drug-loaded prebiotic microcapsules (BBR/CUR@MC) had a significantly longer retention time in the colon than that in the unloaded MC group, which might be due to the wrinkled surface of the microcapsules helped prolong their retention time in the colon (Fig. 4B–F). In summary, the constructed BBR/CUR@MC has good colon targeting and colon retention capacity.

### 3.4 Evaluation of the biosafety of BBR/CUR@MC

**Fig. 5:**
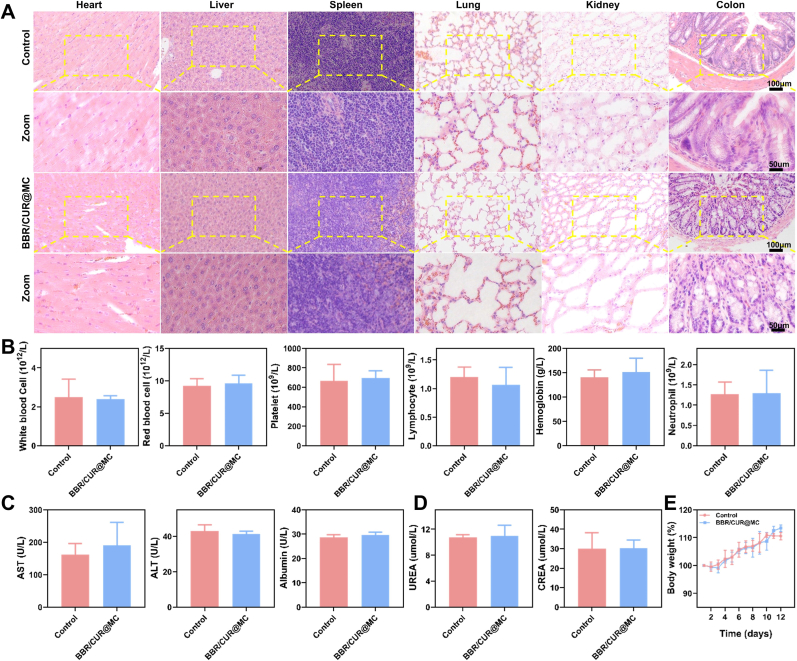
Biosafety evaluation of BBR/CUR@MC. (A) H&E staining results of the heart, liver, spleen, lung, kidney, and colon in mice from each group. (B–D) Results of routine blood tests (B), liver function index tests (ALT: Alanine aminotransferase, AST: Aspartate aminotransferase) (C), and renal function index tests (CREA: Creatinine) (D). (E) Body weight change rate of mice in each group. (n = 3).

Given that the particle size of BBR/CUR@MC far exceeds cellular uptake capacity, in vitro cellular experiments are technically unfeasible. Additionally, the biosafety of its dissociated components has been well-established, and *in vivo* animal study data more closely reflect physiological characteristics while avoiding redundant validation—thus, only animal-based safety experiments were conducted. To evaluate the safety of BBR/CUR@MC administration, short-term and long-term multi-dimensional biosafety verification was performed. Both long-term and short-term histological observation showed that, compared with the healthy control group, the major organs (liver, kidney, heart, spleen, lung) of mice in the BBR/CUR@MC treatment group had intact tissue structures, no obvious pathological changes, normal cell morphology, and no signs of damage such as cellular edema, necrosis, or inflammatory cell infiltration, indicating that BBR/CUR@MC had no significant adverse effects on the structure of important organs (Fig. 5A and S1A). Both long-term and short-term hematological and biochemical tests showed that there were no statistically significant differences in blood routine (white blood cell, red blood cell, platelet, lymphocyte, hemoglobin, neutrophil), liver function (AST, ALT, albumin), and kidney function (UREA, CREA) indices between the BBR/CUR@MC treatment group and the healthy control group (Fig. 5B–D and S1B-D), confirming that BBR/CUR@MC had no toxicity to the blood, liver, and kidneys. In addition, the weight gain trends of the two groups were basically consistent during the experiments, with no significant differences (Fig. 5E). In conclusion, BBR/CUR@MC has good biosafety, providing a feasible basis for its *in vivo* experimental application.

### 3.5 Therapeutic efficacy of BBR/CUR@MC in UC is superior to single-drug microcapsule formulations

**Fig. 6:**
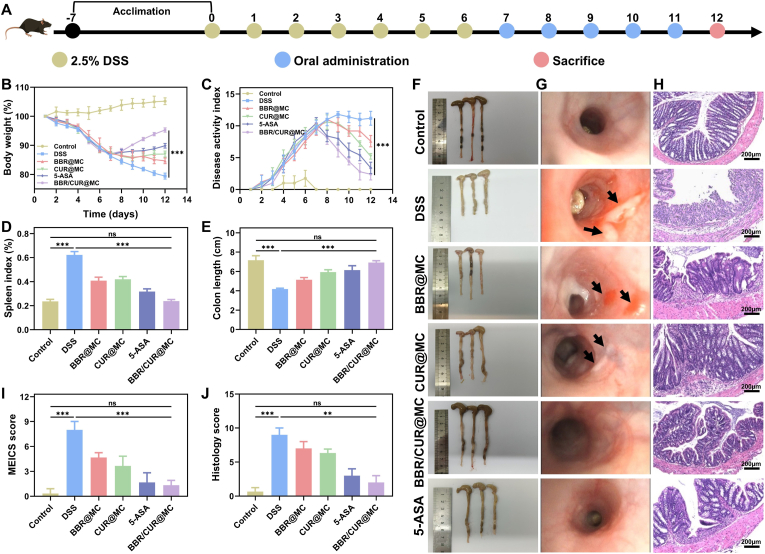
Therapeutic effects of BBR/CUR@MC on experimental colitis. (A) Schematic diagram of the establishment and treatment of the experimental colitis mouse model. (B and C) Body weight (B) and disease activity index (C) of mice during the experiment (n = 5). (D and E) Spleen index and statistical results of colon length in each group of mice at the end of the experiment (n = 5). (F–J) Colon appearance (F), endoscopic examination (G), H&E staining (H), MEICS scores (I), and histological scores (J) of mice. (n = 3; *∗∗P* < 0.01, *∗∗∗P* < 0.001).

To systematically evaluate the therapeutic effect of BBR/CUR@MC, a colitis model was established: mice were given 2.5 % DSS for 7 consecutive days to induce acute colitis, and after successful modeling, mice were treated with normal saline, 100 mg/kg 5-ASA, BBR@MC, CUR@MC, or BBR/CUR@MC for the next 5 consecutive days (Fig. 6A). At the end of the experiments, the mice were euthanized, and therapeutic efficacy was comprehensively evaluated through multiple indicators. After DSS induction, the model mice showed typical colitis characteristics: significant weight loss within 7 days, increased disease activity index (DAI), and elevated spleen index (Fig. 6B–D). After 5 days of drug treatment, weight loss, increased DAI, and elevated spleen index were improved in all groups, with the most significant relief in the BBR/CUR@MC group (Fig. 6B–D). Colon length assessment also showed that BBR/CUR@MC was more effective than 5-ASA and single-drug microcapsule groups in colitis (Fig. 6E and F). The results of H&E staining and endoscopic examination showed that the DSS model group had colonic ulcers of varying sizes, mucosal bleeding, and blurred vascular texture (Fig. 6G). After treatment with BBR@MC and CUR@MC, the colonic mucosal ulcers in the mice healed, and MEICS scores decreased. However, a small number of bleeding points and vascular abnormalities remained in the colon (Fig. 6G and I). In contrast, after receiving BBR/CUR@MC treatment, the colonic mucosal ulcers achieved endoscopic healing, vascular texture was clear, and MEICS scores showed no significant difference from the normal control group (Fig. 6G and I). H&E staining results showed that the BBR/CUR@MC group had significantly reduced inflammatory cell infiltration, restored orderly arrangement of epithelial cells, and significantly decreased histological scores (Fig. 6H and J). In summary, BBR/CUR@MC has a significant therapeutic effect on colitis, and the combined application of BBR and CUR is superior to single-drug treatment.

### 3.6 Intestinal repair effect of BBR/CUR@MC in UC is stronger than single-drug microcapsule formulations

**Fig. 7:**
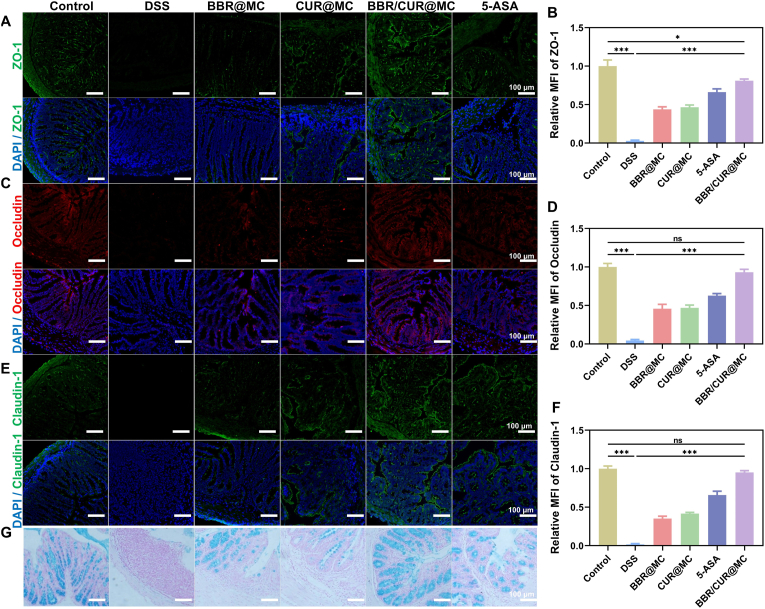
The repair effect of BBR/CUR@MC on the intestinal barrier. (A and B) Immunofluorescence staining results (A) and statistical analysis of mean fluorescence intensity (B) of ZO-1 in colon tissues of mice in each experimental group. (C and D) Immunofluorescence staining results (C) and statistical analysis of mean fluorescence intensity (D) of Claudin-1. (E and F) Immunofluorescence staining results (E) and statistical analysis of mean fluorescence intensity (F) of Occludin. (G) Alcian blue staining results of mouse colon tissues. (n = 3; *∗P* < 0.05, *∗∗∗P* < 0.001). (For interpretation of the references to colour in this figure legend, the reader is referred to the Web version of this article.)

Inflammatory cascades in UC originate from intestinal barrier impairment, making barrier restoration a critical therapeutic target [23]. To assess the protective capacity of BBR/CUR@MC on the colonic epithelial barrier, the expression of tight junction (TJ) proteins (ZO-1, Occludin, and Claudin-1) was evaluated in colonic tissues of the treated murine models. Immunofluorescence staining showed that compared with the control group, TJ proteins expression was markedly downregulated in the DSS induction group, suggesting intestinal barrier damage (Fig. 7A–F). After gavage of 5-ASA, BBR@MC, CUR@MC, or BBR/CUR@MC, the expression levels of ZO-1, Occludin, and Claudin-1 in murine colonic tissues were partially rescued, indicating that the intestinal barrier function was partially recovered. In addition, the most pronounced restoration was observed in the BBR/CUR@MC group (Fig. 7A–F). To gain deeper insights into the effects of BBR/CUR@MC on the intestinal mucus barrier, Alcian blue and periodic acid-Schiff (PAS) staining were performed on colonic tissue sections. Notably, compared with the control group, the DSS-induced group showed a significant reduction in the number of goblet cells and secretory vesicles, resulting in marked impairment of intestinal mucus secretion capacity (Fig. 7G and S2). In contrast, all treatment groups exhibited a partial increase in goblet cell counts within the colonic mucosa, which was consistent with the partial restoration of intestinal mucus barrier function (Fig. 7G and S2). Among these, the BBR/CUR@MC group achieved the most prominent restoration of goblet cell numbers (Fig. 7G and S2). In conclusion, the colitis model induced by DSS exhibited damage to the intestinal barrier and intestinal mucosa, while treatment with BBR/CUR@MC could significantly repair this damage.

### 3.7 BBR/CUR@MC alleviates colonic inflammation by "top-down" inhibiting inflammatory cascades and "full-cycle" reducing oxidative damage

**Fig. 8:**
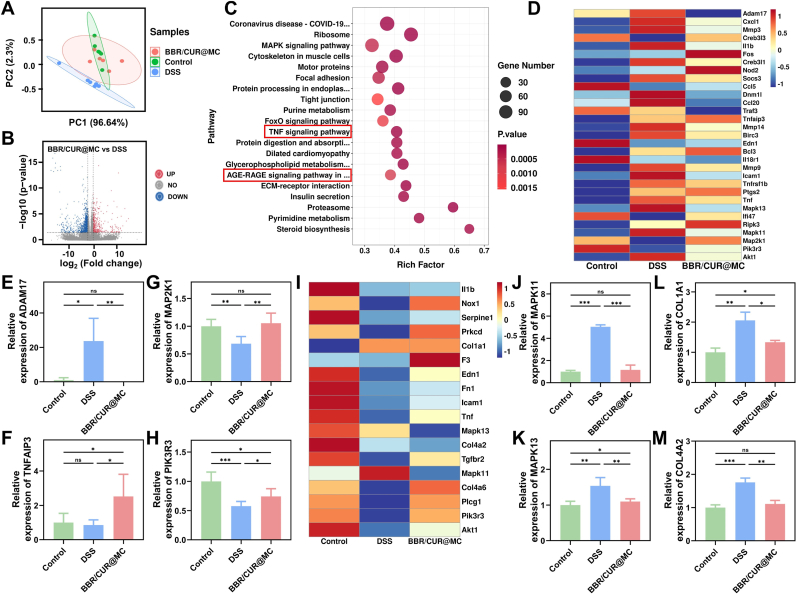
Transcriptomic analysis of the therapeutic effect of BBR/CUR@MC. (A) Principal component analysis of the control group, DSS group, and BBR/CUR@MC group. (B) Volcano plot of differential genes between the DSS group and the BBR/CUR@MC group (Red dots: FC > 2 and q < 0.05, Blue dots: FC < 0.5 and q < 0.05, Gray dots: do not meet the screening criteria). (C) Bubble plot of KEGG enrichment analysis for the common differentially expressed genes among the three groups. (D) Expression heatmap of TNF signaling pathway-related genes in the three groups. (E–H) Statistical analysis of relative expression levels of ADAM17 (E), TNFAIP3 (F), MAP2K1 (G), and PIK3R3 (H). (I) Expression heatmap of AGE-RAGE signaling pathway-related genes in the three groups. (J–M) Statistical analysis of relative expression levels of MAPK11 (J), MAPK13 (K), COL4A1 (L), and COL1A1 (M). (n = 5; *∗P* < 0.05, *∗∗P* < 0.01, *∗∗∗P* < 0.001). (For interpretation of the references to colour in this figure legend, the reader is referred to the Web version of this article.)

To explore the synergistic mechanism of BBR/CUR@MC in UC treatment, transcriptome analysis was performed on colonic tissues from the Control, DSS, and BBR/CUR@MC groups. Principal component analysis (PCA) results showed that the gene expression pattern of the BBR/CUR@MC group was highly similar to that of the control group, while the DSS group showed significant differences from the other two groups (Fig. 8A), suggesting that DSS treatment significantly altered the gene expression pattern in mice, and BBR/CUR@MC treatment could restore the gene expression trend to a normal state. Differential genes were screened based on the criteria of FC (Fold Change) > 2 or < 0.5 and q < 0.05, and the results showed that compared with the DSS group, 1259 genes were significantly downregulated and 379 genes were significantly upregulated in the BBR/CUR@MC group (Fig. 8B). KEGG analysis of common differentially expressed genes among the three groups confirmed that BBR/CUR@MC exerted therapeutic effects by regulating the TNF signaling pathway and AGE-RAGE signaling pathway in diabetic complications (Fig. 8C).

Heatmap analysis of differential genes in the TNF signaling pathway suggested that BBR/CUR@MC exerted anti-inflammatory effects by inhibiting this pathway (Fig. 8D). Further gene statistical analysis showed that BBR/CUR@MC can inhibit ADAM17, blocking the initiation of the TNF signaling pathway at the source (Fig. 8E). Meanwhile, it activated TNFAIP3, PIK3R3, and MAP2K1 to regulate NF-κB and MAPK activity, reducing the amplification of downstream inflammatory responses (Fig. 8F–H). Heatmap analysis of differential genes in the AGE-RAGE signaling pathway (Fig. 8I) and related gene statistics showed that BBR/CUR@MC can negatively feedback-block upstream pro-oxidative signals by inhibiting the expression of MAPK11, MAPK13, COL4A1, and COL1A1. Furthermore, CUR released from BBR/CUR@MC in the intestinal tract directly scavenges ROS. Together with the aforementioned inhibition of *de novo* ROS generation, these two effects constitute a "full-cycle" antioxidant mechanism—i.e., scavenging preformed ROS and suppressing *de novo* ROS production—thereby mitigating oxidative damage (Fig. 8J–M). Subsequently, we conducted ELISA to further quantify the protein expression levels of the key targets (ADAM17, TNFAIP3, MAPK11, and COL1A1) in the TNF and AGE-RAGE signaling pathways. The results of this protein-level analysis were consistent with those of the transcriptional level analysis, thereby validating the reliability of BBR/CUR@MC in blocking TNF signaling to activate and inhibiting AGE-RAGE signaling (Fig. S3A–D). In summary, BBR/CUR@MC synergistically alleviates colonic inflammation by "top-down" inhibiting inflammatory cascades and "full-cycle" reducing oxidative damage.

### 3.8 BBR/CUR@MC exhibits superior anti-inflammatory and antioxidant activity in UC compared to single-drug microcapsules

**Fig. 9:**
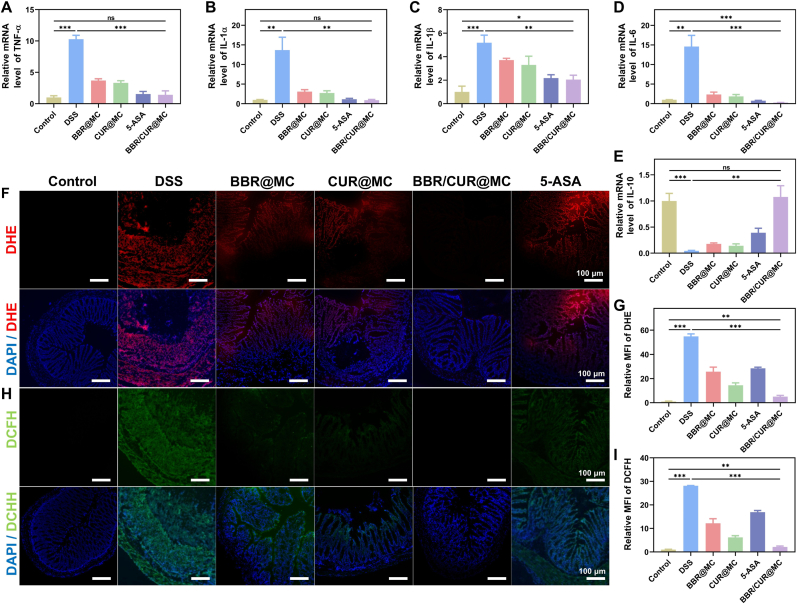
Anti-inflammatory and antioxidant capacities of BBR/CUR@MC in colitis. (A–E) mRNA transcription levels of inflammatory cytokines, including TNF-α (A), IL-1α (B), IL-1β (C), IL-6 (D), and IL-10 (E) in the colon of mice in each group. (F and G) DCFH-DA fluorescent staining results (F) and statistical analysis of mean fluorescence intensity (G) of mouse colon tissues in each group. (H and I) DHE fluorescent staining results (H) and statistical analysis of mean fluorescence intensity (I) of mouse colon tissues in each group. (n = 3; *∗P* < 0.05, *∗∗P* < 0.01, *∗∗∗P* < 0.001).

Ultimately, we assessed the anti-inflammatory and antioxidant potential of BBR/CUR@MC in the context of ulcerative colitis. RT-qPCR detection of colon homogenates from DSS-induced mice showed that the transcription level of pro-inflammatory cytokines such as TNF-α, IL-1α, IL-1β, and IL-6 was significantly upregulated in the DSS group, while the transcription level of the anti-inflammatory cytokine IL-10 was significantly downregulated (Fig. 9A–E). After drug treatment, this imbalance of inflammatory factors was reversed, with the BBR/CUR@MC group showing a significantly better improvement than the single-drug microcapsule groups (Fig. 9A–E). To assess antioxidant activity, ROS in colonic tissues were labeled using DCFH-DA and DHE probes. Quantitative analysis of mean fluorescence intensity revealed that the DSS-induced group exhibited the strongest fluorescence signals, reflecting excessive ROS accumulation and marked oxidative injury (Fig. 9F–I). In contrast, the BBR@MC and 5-ASA groups showed attenuated fluorescence, and notably, the BBR/CUR@MC group displayed the weakest fluorescence intensity, underscoring its superior ROS-scavenging efficacy compared to all other experimental cohorts. In summary, BBR/CUR@MC exhibits superior anti-inflammatory and antioxidant activity in UC compared to single-drug microcapsules.

## 4 Discussion

A central driver of UC pathogenesis is the self-perpetuating "oxidative stress-inflammation" vicious cycle [24,25]. This cycle is sustained through intricate crosstalk between the TNF signaling pathway and the AGE-RAGE signaling pathway (originally characterized in diabetic complications). Following initial intestinal barrier disruption, microbial products and other stimuli activate immune cells, which release proinflammatory cytokines such as TNF-α via the TNF signaling pathway to initiate inflammatory cascades. TNF-α dualistically activates downstream pathways, including NF-κB and MAPK—promoting production of additional proinflammatory mediators (e.g., IL-1β, IL-6) and recruiting infiltrating immune cells such as neutrophils [[26], [27], [28]]—while inducing NADPH oxidase expression to trigger excessive ROS generation, thereby initiating oxidative stress [[29], [30], [31]]. Accumulated ROS further activates the AGE-RAGE signaling pathway, accelerating the formation of advanced glycation end products (AGEs) [32]. AGE binding to the receptor RAGE reactivates MAPK and NF-κB signaling, amplifying inflammatory signals (via increased proinflammatory cytokine secretion) and exacerbating ROS production to establish a secondary amplifying loop [[33], [34], [35]]. This synergistic interplay between the two pathways perpetuates the vicious cycle, ultimately driving recurrent intestinal mucosal damage, ulceration, and UC chronification.

ADAM17, a metalloproteinase, cleaves membrane-bound TNF-α to release its soluble form, facilitating receptor binding and thus acting as an upstream activator initiating the TNF signaling pathway [[36], [37], [38]]. Conversely, TNFAIP3 inhibits key NF-κB pathway molecules to block TNF-α-induced inflammatory signal amplification, mediating negative feedback regulation [39,40]. As a PI3K regulatory subunit, PIK3R3 activates the PI3K/Akt pathway to suppress excessive NF-κB activation, thereby attenuating proinflammatory cytokine release mediated by this pathway [41,42]. Meanwhile, MAP2K1 functions as an upstream kinase in the MAPK pathway to activate ERK1/2, participating in inflammatory signal transduction; upon moderate activation, it can also negatively regulate NF-κB activity via feedback mechanisms [43]. Through coordinated upstream-downstream interactions, these four molecules collectively modulate the activation intensity and balance of the TNF signaling pathway, influencing the duration and magnitude of inflammatory cascades. In this study, BBR/CUR@MC inhibits ADAM17 to block pathway initiation, while activating TNFAIP3, PIK3R3, and MAP2K1—attenuating downstream inflammatory amplification by regulating NF-κB and MAPK activity, thereby achieving "top-down" attenuation of cascading inflammation.

Within the AGE-RAGE signaling pathway, MAPK11 and MAPK13—members of the p38 MAPK family—are activated to exacerbate ROS production and inflammation by enhancing NADPH oxidase activity and promoting proinflammatory cytokine transcription [35,44]. Aberrant expression of COL4A1 and COL1A1 leads to their oxidative modification products, which accelerate AGE deposition and drive tissue damage [45]. BBR/CUR@MC inhibits these four molecules, reducing ROS generation and AGE binding sites, thereby establishing negative feedback to block upstream pro-oxidative signals. Combined with CUR's direct ROS-scavenging capacity, these effects collectively form a "full-cycle" antioxidant mechanism that mitigates oxidative damage.

Current co-delivery systems for natural bioactive compounds (e.g., nanoparticles, polymeric micelles, conventional hydrogels), while enhancing drug solubility and bioavailability, suffer from inherent limitations. For instance, nanoparticle-based systems are often plagued by poor colloidal stability and inadequate targeting [46]. In contrast, BBR/CUR@MC achieves superior colonic targeting and retention via pH-responsive chitosan-alginate coatings and wrinkled surface topography. Polymeric micelles merely enhance bioavailability by improving solubility but exhibit poor efficacy in synergistic therapy [47]. BBR/CUR@MC, however, innovatively enables "carrier-drug synergy"—regulating the intestinal microenvironment, co-delivering BBR/CUR, and targeting TNF and AGE-RAGE signaling pathways to break the inflammation-oxidative stress vicious cycle—outperforming polymeric micelles' monofunctionality. Conventional hydrogels exhibit poor drug release control and short colonic residence [48]. BBR/CUR@MC leverages microfluidic electrospray for uniform particle size and stimuli-responsive release, and its chitosan outer layer enhances mucus adhesion to prolong colonic retention. Collectively, BBR/CUR@MC is not a mere optimization of existing systems but a three-dimensional platform integrating colonic targeting, intestinal microenvironment modulation, and synergistic drug delivery. It specifically addresses these systems’ core limitations, yielding a multifunctional co-delivery platform better tailored to ulcerative colitis therapy.

## 5 Conclusion

This study aims to construct prebiotic microcapsules (BBR/CUR@MC) for the co-delivery of BBR and CUR and explore their potential mechanism in treating ulcerative colitis (UC). First, network pharmacology preliminarily predicted that BBR and CUR have the potential to synergistically regulate the TNF and AGE-RAGE signaling pathways, exerting UC therapeutic effects by inhibiting inflammatory cascades and synergistic antioxidant activity. On this basis, BBR/CUR@MC was successfully constructed using microfluidic electrospray technology, and evaluation confirmed its good colon targeting and biosafety. In DSS-induced mouse colitis models, oral administration of BBR/CUR@MC exerts therapeutic effects through dual mechanisms: on one hand, it regulates TNF signaling pathway targets such as ADAM17 to "top-down" inhibit UC inflammatory cascades; on the other hand, it achieves "full-cycle" antioxidant activity by directly scavenging ROS and inhibiting AGE-RAGE signaling pathway targets such as MAPK11/13. These two mechanisms jointly repair the intestinal barrier and alleviate colitis symptoms. In summary, this study provides a potential multi-mechanism therapeutic scheme for UC, which is expected to bring new insights into UC treatment.

## CRediT authorship contribution statement

**Huanyu Li:** Writing – original draft, Validation, Software, Methodology, Investigation, Formal analysis, Data curation. **Chuanyu Zhang:** Writing – review & editing, Investigation, Funding acquisition. **Ziwei Yang:** Writing – review & editing. **Yifan Li:** Data curation, Investigation, Methodology. **Dan Liu:** Supervision, Resources, Project administration, Funding acquisition, Conceptualization. **Yanan Zhang:** Methodology, Formal analysis, Data curation. **Lingmin Zhang:** Methodology, Formal analysis, Conceptualization. **Ning Wang:** Investigation, Data curation. **Mingxin Zhang:** Data curation. **Mingzhen Zhang:** Data curation. **Zhaoxiang Yu:** Resources, Methodology, Investigation, Conceptualization. **Xueyong Wei:** Writing – review & editing, Supervision, Resources, Project administration, Funding acquisition, Conceptualization. **Yujie Zhang:** Writing – review & editing, Methodology, Funding acquisition, Formal analysis, Conceptualization.

## Availability of data and materials

All data generated and analyzed in the present study are contained within this published article and its accompanying supplementary materials. For the datasets utilized or analyzed herein, access can be obtained by making a reasonable request.

## Funding

This work was supported by the 10.13039/501100001809National Natural Science Foundation of China (82502548), the National Key Research and Development R&D Program of China (2022YFC2406600) and the Program for Innovation Team of Shaanxi Province (2021TD-23), the Shaanxi Province Key Research and Development Program (2023-YBSF-072 and 2024JC-YBMS-664), and the Xi'an Science and Technology Plan Project (24YXYJ0143).

## Declaration of competing interest

The authors declare that they have no known competing financial interests or personal relationships that could have appeared to influence the work reported in this paper.

## Data Availability

Data will be made available on request.
