# Supplementary material for: Engineered prebiotic microcapsules Co-Encapsulating berberine and curcumin Elicit multi-synergistic therapy for ulcerative colitis
Source: Mater Today Bio. 2026 Jan 7;37:102778. doi: 10.1016/j.mtbio.2026.102778 (PMC12828602; doi:10.1016/j.mtbio.2026.102778)
Supplement: Multimedia component 1 [file mmc1.docx]

**Engineered Prebiotic Microcapsules Co-Encapsulating Berberine and Curcumin Elicit Multi-Synergistic Therapy for Ulcerative Colitis**

Huanyu Li^1,2#^, Chuanyu Zhang^3,4#^, Ziwei Yang^3,4#^, Yifan Li,^5^ Dan Liu^6^, Yanan Zhang^7^, Lingmin Zhang^8^, Ning Wang^9^, Mingxin Zhang^7^, Mingzhen Zhang^1^, Zhaoxiang Yu^7*^, Xueyong Wei^3,4*^, and Yujie Zhang^1*^

Affiliations:

1. Institute of Translational Medicine, School of Basic Medical Sciences, Xi’an Jiaotong University, Xi’an, Shaanxi, 710061, China
2. Department of Laboratory Medicine, Chongqing Center for Clinical Laboratory, Chongqing Academy of Medical Sciences, Chongqing General Hospital, School of Medicine, Chongqing University, Chongqing, 401147, China.
3. School of Instrument Science and Technology, Xi’an Jiaotong University, Xi’an, Shaanxi, 710049, China
4. State Key Laboratory for Manufacturing Systems Engineering, Xi'an Jiaotong University, Xi’an, Shaanxi, 710049, China
5. Second Clinical Medical College, Shaanxi University of Chinese Medicine, Xianyang, Shaanxi, 712046, China.
6. State Key Laboratory of Holistic Integrative Management of Gastrointestinal Cancers and National Clinical Research Center for Digestive Diseases, Xijing Hospital of Digestive Diseases, Fourth Military Medical University, Xi’an, Shaanxi,710032, China
7. The First Affiliated Hospital of Xi’an Medical University, Xi’an, Shaanxi, 710077, China
8. Department of Anesthesiology, The First Affiliated Hospital of Xi'an Jiaotong University, Xi’an, Shaanxi, 710061, China
9. Department of Anaesthesiology, The Second Affiliated Hospital, Xi'an Jiaotong University, Xi’an, Shaanxi, 710004, China

# These authors contributed equally to this article.

* Correspondence authors

Yujie Zhang (ORCID: 0000-0002-7969-234X)

Email address: zhangyujie@xjtu.edu.cn

Xueyong Wei (ORCID: 0000-0002-6443-4727)

Email address: seanwei@mail.xjtu.edu.cn

Zhaoxiang Yu (ORCID: 0009-0005-0215-1878)

Email address: yuzhaoxiang@xiyi.edu.cn

**Supplementary Figure**

**
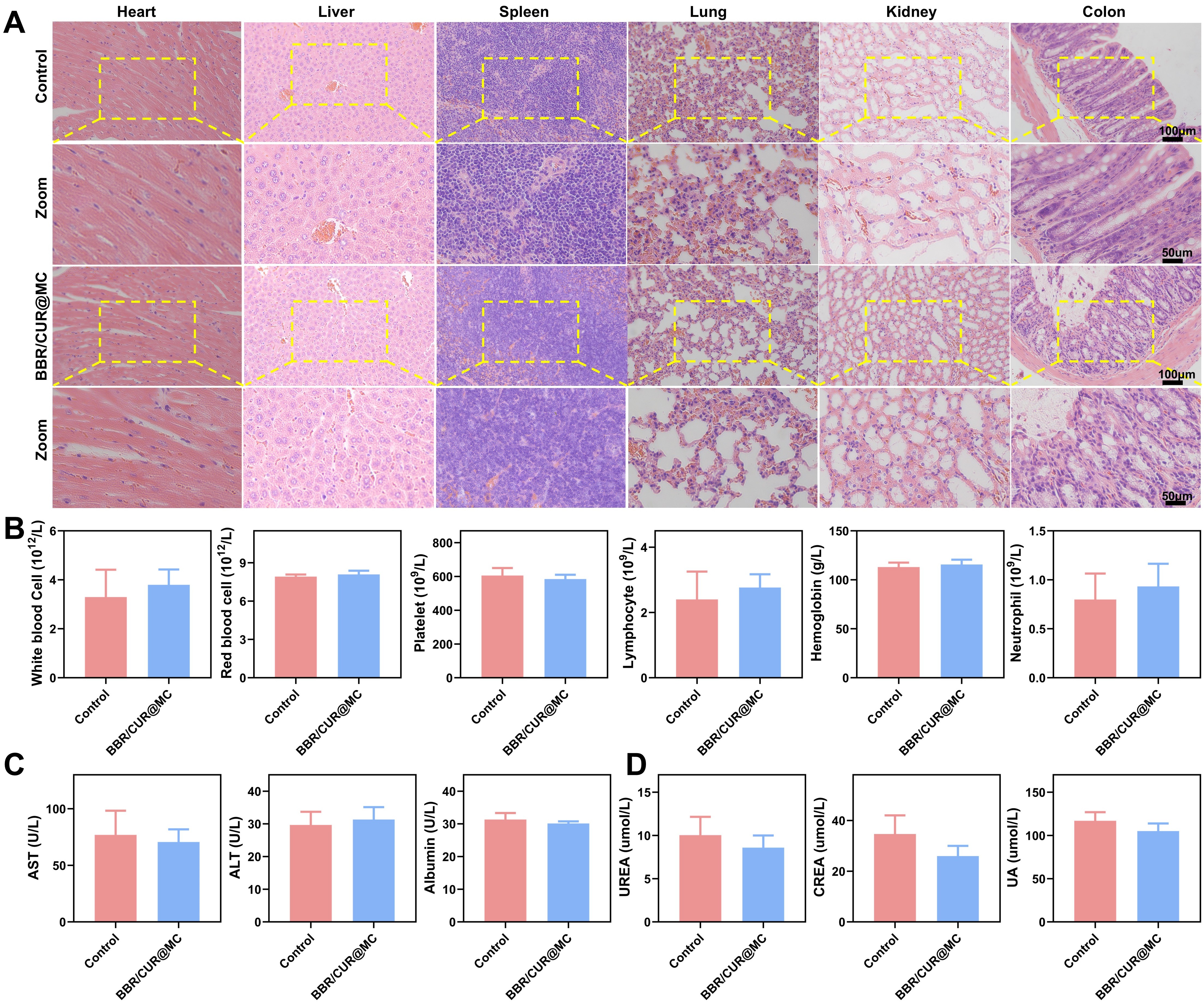
**

**Figure S1. Biosafety evaluation of BBR/CUR@MC.** (A) H&E staining results of the heart, liver, spleen, lung, kidney, and colon in mice from each group. (B-D) Results of routine blood tests (B), liver function index tests (ALT: Alanine aminotransferase, AST: Aspartate aminotransferase) (C), renal function index tests (CREA: Creatinine, UA: Uric acid ) (D). (n = 3).

**
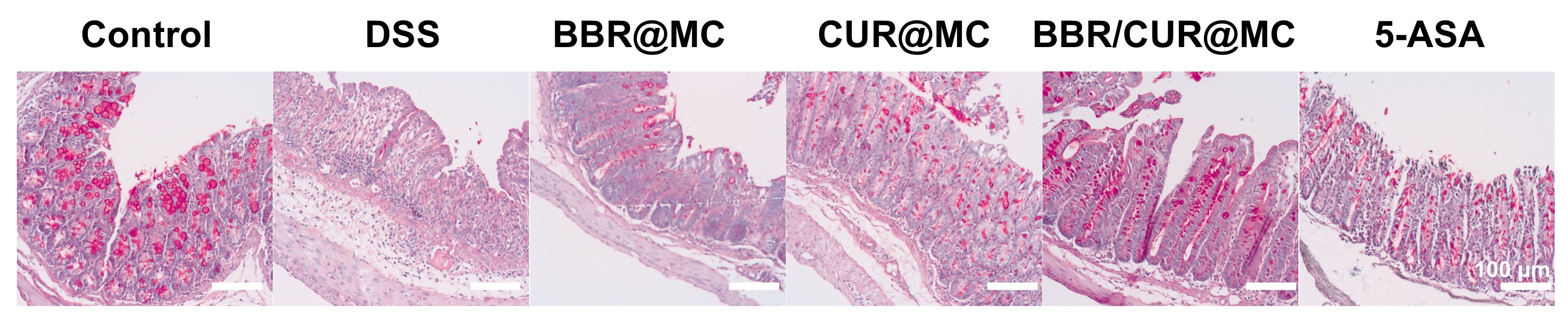
**

**Figure S2.** Periodic acid-Schiff (PAS) staining results of mouse colon tissues.

**
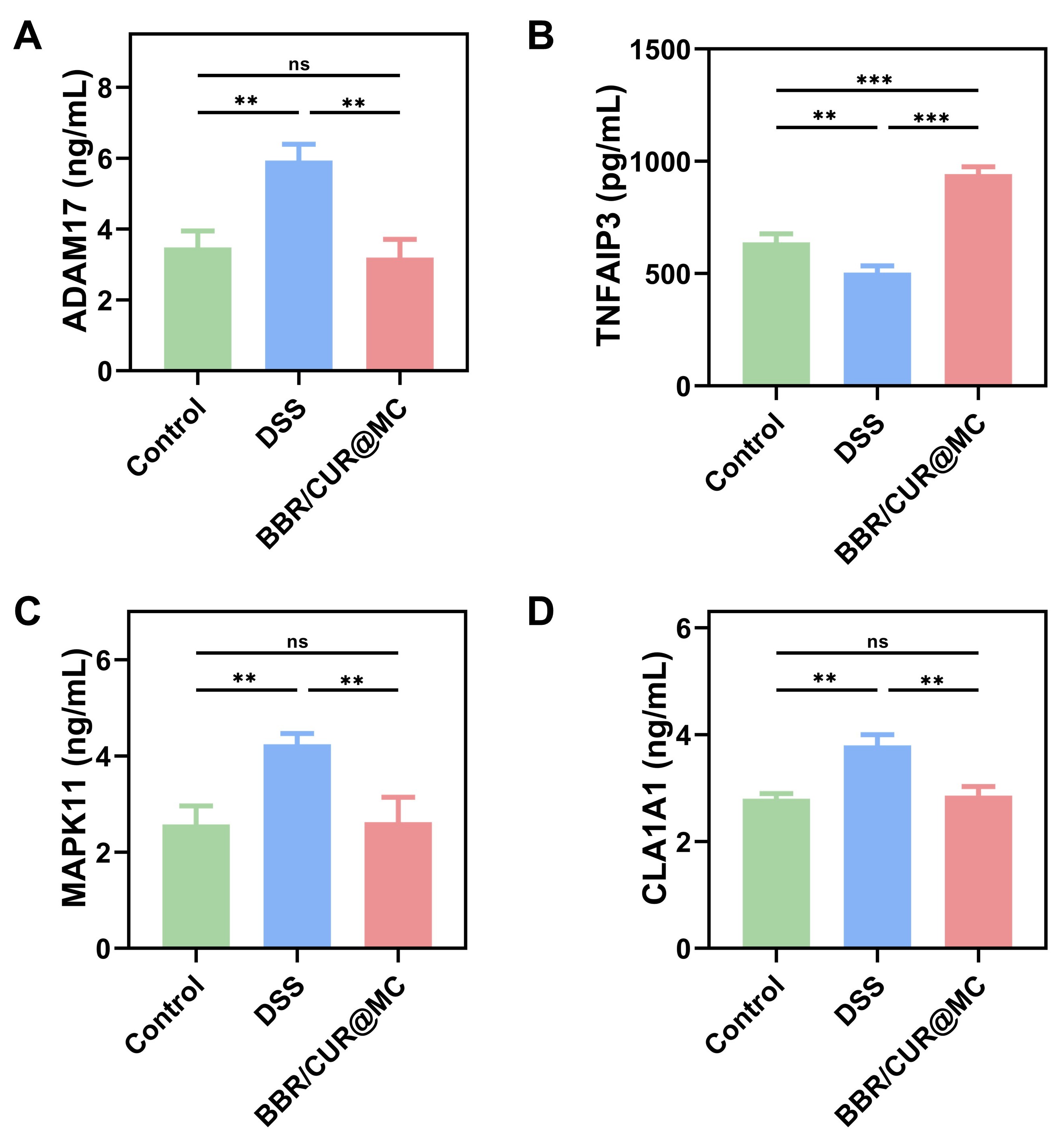
**

**Figure S3.** Protein expression levels of (A) ADAM17, (B) TNFAIP3, (C) MAPK11, and (D) COL1A1.

**Supplementary Table 1.** Detailed criteria for the Disease Activity Index (DAI) scoring

| **Score** | **Percentage of Weight Loss** | **Stool Consistency** | **Fecal Occult Blood** |
| --- | --- | --- | --- |
| 0 | 0 | Normal stool | Negative |
| 1 | 1%-5% | Soft pasty stool | Positive fecal occult blood or a small amount of blood streaks |
| 2 | 5% - 10% | Loose unformed stool | Obvious blood in stool |
| 3 | 10% - 20% | Watery stool | Severe bloody stool |
| 4 | >20% | - | - |

**Supplementary Table 2.** Detailed criteria for Mouse Endoscopic Index of Colitis Severity (MEICS) scoring

| **Item** | **Score Criteria** | **Score Range** |
| --- | --- | --- |
| Thickening of the colon | 0: Transparent; 1: Moderate; 2: Marked; 3: Non-transparent | 0–3 |
| Changes in the vascular pattern | 0: Normal; 1: Moderate; 2: Marked; 3: Bleeding | 0–3 |
| Fibrin visible | 0: None; 1: Little; 2: Marked; 3: Extreme | 0–3 |
| Granularity of the mucosal surface | 0: None; 1: Moderate; 2: Marked; 3: Extreme | 0–3 |
| Stool consistency | 0: Normal + solid; 1: Still shaped; 2: Unshaped; 3: Spread | 0–3 |

**Supplementary Table 3.** Colonic histological scoring

| **Scoring Dimension** | **Criteria for 0 Points** | **Criteria for 1 Point** | **Criteria for 2 Points** | **Criteria for 3 Points** |
| --- | --- | --- | --- | --- |
| Inflammatory Cell Infiltration | The cellular composition of the intestinal mucosal lamina propria is normal, with no signs of inflammatory cell infiltration. | A small number of inflammatory cells (such as lymphocytes, monocytes, etc.) are scattered in the mucosal lamina propria, basically not affecting the normal morphology of the tissue. | The number of inflammatory cells increases significantly, distributed in foci or small patches, and neutrophils, eosinophils, etc., may appear, which has caused a certain degree of damage to the intestinal tissue. | A large number of inflammatory cells diffusely infiltrate the mucosal lamina propria, leading to obvious widening of the tissue interstitium, disordered cell arrangement, and severe destruction of the normal tissue structure. |
| Epithelial Changes | Epithelial cells have regular morphology and complete structure; the number of goblet cells is sufficient with normal morphology, and the cells are arranged closely and orderly. | Epithelial cells are mildly swollen, some cells are slightly disorganized, and the number of goblet cells is slightly reduced, but the overall impact is minor. | The swelling of epithelial cells is aggravated, the cell gaps are obviously widened, the cell nuclei show pyknosis, the number of goblet cells is reduced to about 50%, and the integrity of the epithelial tissue is greatly affected. | A large number of epithelial cells are necrotic and detached, the surface of the intestinal mucosa is incomplete, goblet cells almost completely disappear, and the epithelial tissue is severely damaged. |
| Mucosal Structure | The intestinal mucosal structure is intact; crypts have regular morphology, uniform size, and are neatly and orderly arranged; the height and morphology of villi are within the normal range. | Crypts show mild deformation, individual crypts are branched or irregular in shape, and villi are slightly shortened or widened. | The structure of crypts is obviously disorganized; some crypts are fused or have disappeared, and villi are significantly shortened, thickened, and fused. | The mucosal structure is severely damaged, a large number of crypts are missing, villi almost disappear, and are replaced by granulation tissue or fibrous tissue proliferation, and the normal function of the intestinal mucosa is seriously impaired. |

**Supplementary Table 4.** Sequences of the primers used for qRT-PCR

| **Gene** | **Forward sequences (5’-3’)** | **Reverse sequences (5’-3’)** |
| --- | --- | --- |
| TNF-α | AGGCTGCCCCGACTACGT | CATCAGAGGCAAGGAGGAAAAC |
| IL-1α | GTATGCCTACTCGTCGGGAGGAG | GCAACACGGGCTGGTCTTCTC |
| IL-1β | TCGCTCAGGGTCACAAGAAA | CATCAGAGGCAAGGAGGAAAAC |
| IL-6 | ACAAGTCGGAGGCTTAATACACAT | TTGCCATTGCACAACTCTTTTC |
| IL-10 | GGTTGCCAAGCCTTATCGGA | CTTCTCACCCAGGGAATTCA |
